# Dihydroquercetin Activates AMPK/SIRT1/PGC-1α Signaling to Attenuate Arsenic Trioxide-Induced Apoptosis and Ferroptosis in AC16 Cells and HUVECs

**DOI:** 10.3390/ijms27198753

**Published:** 2026-09-30

**Authors:** Wenlei Zhang, Rui Duan, Xinsheng Duan, Sijing Tao, Haoxuan Li, Jinyan Li, Xinru Wang, Lijuan Yue, Kai Ren, Tianyu Zhao, Meihua Guo, Xiaoxiang Guan, Xin Hai

**Affiliations:** Department of Pharmacy, First Affiliated Hospital of Harbin Medical University, 23 YouZheng Str, Nangang District, Harbin 150007, China13523229801@163.com (R.D.);

**Keywords:** dihydroquercetin, arsenic trioxide, cardiotoxicity, apoptosis, ferroptosis, network pharmacology

## Abstract

Arsenic trioxide (ATO) is a first-line therapeutic agent for acute promyelocytic leukemia (APL), but its clinical utility is constrained by dose-dependent cardiotoxicity. This study aimed to systematically evaluate the cytoprotective potential of dihydroquercetin (DHQ), a natural flavonoid derived from coniferous species with multimodal antioxidant properties, against ATO-induced cellular injury. An integrated combining network pharmacology with experimental validation was employed to elucidate the underlying molecular mechanisms. Cardiotoxicity models were established using AC16 human cardiomyocytes and human umbilical vein endothelial cells (HUVECs). Cytotoxicity, reactive oxygen species (ROS), mitochondrial membrane potential, DNA damage (γ-H2AX), apoptosis (TUNEL, Bcl-2, Bax, Caspase-3), and ferroptosis markers (GPX4, xCT, intracellular iron) were assessed. The AMPK/SIRT1/PGC-1α axis was validated using Compound C. ATO exposure induced significant cytotoxicity, oxidative stress, mitochondrial dysfunction, and activated both apoptosis and ferroptosis pathways. Co-treatment with DHQ dose-dependently attenuated these injuries. Mechanistically, DHQ activated AMPK/SIRT1/PGC-1α, increasing phosphorylated AMPK, SIRT1, and PGC-1α. Compound C abrogated DHQ-mediated pathway activation and its protection against oxidative stress, apoptosis, and ferroptosis. DHQ protects against ATO cardiotoxicity by activating the AMPK/SIRT1/PGC-1α pathway, thereby reducing oxidative stress and concurrently inhibiting apoptosis and ferroptosis. These findings position DHQ as a promising candidate for protecting cardiomyocytes and endothelial cells from ATO-induced injury.

## 1. Introduction

Arsenic trioxide (ATO), pharmacologically known as Pi-Shuang, modern oncology repurposed this historical toxin through pioneering work by Chinese hematologists in the 1970s [1]. The seminal development of the “Ailing Yihao” regimen marked a therapeutic revolution, establishing ATO as the first-line agent for acute promyelocytic leukemia (APL) through dual molecular mechanisms: PML-RARα oncoprotein degradation and caspase-mediated apoptosis initiation [2,3]. Furthermore, existing studies have shown that arsenic trioxide can inhibit various solid tumors, have antiviral effects, and improve systemic sclerosis and systemic lupus erythematosus [4,5,6,7]. However, the toxic side effects caused by arsenic trioxide have limited its clinical application. Clinical data shows, despite transforming APL prognosis (5-year survival >90%), ATO’s clinical application remains constrained by dose-dependent cardiotoxicity [8,9].

ATO induces dose-dependent remodeling of myocardial tissue structure and ultrastructure [10]. Clinically, it prolongs the QT interval and predisposes to ventricular reentrant tachycardia [11]. Pathologically, it promotes myocardial fibrosis, hypertrophy, programmed cell death, and systolic dysfunction, in addition to endothelial injury [11,12]. Ample evidence confirms that arsenic exposure is cardiotoxic, largely through programmed cell death in the cardiac microenvironment, including apoptosis, ferroptosis, and autophagy [13,14]. These interconnected mechanisms demonstrate intricate crosstalk [15]. For instance, BID knockout in neuronal cells preserves mitochondrial integrity and protects against both ferroptosis and oxytosis [16]. LINC00618 promotes apoptosis by elevating BAX and cleaved caspase-3 [17]. Sodium nitroprusside inhibits apoptosis, ferroptosis, and inflammation, thereby alleviating nanoplastic toxicity [18]. These findings underscore the therapeutic promise of multi-pathway intervention. However, the spatiotemporal coordination between apoptosis and ferroptosis in ATO-induced cardiotoxicity remains poorly defined. Consequently, clinical strategies to mitigate these side effects have achieved little success, urging the discovery of multi-target inhibitors that can disrupt synergistic cell death mechanisms.

Dihydroquercetin (DHQ), a flavonoid abundant in *Larix sibirica*, *L. gmelinii*, and *Pseudotsuga menziesii* bark (https://www.worldfloraonline.org/search?query=Dihydroquercetin, accessed on 27 July 2026), has emerged as a multifunctional cardioprotective agent [19,20]. It directly scavenges ROS via phenolic hydroxyl-mediated redox cycling, suppresses NF-κB/NLRP3 inflammasome activation, and preserves mitochondrial integrity by stabilizing complex I and inhibiting mPTP [21]. Dihydroquercetin can restore the Sirt1/Nrf2/HO-1 signaling pathway in the heart, and it has a significant cardioprotective effect on the heart damage caused by 5-fluorouracil [22]. It also attenuates myocardial ischemia/reperfusion injury through PI3K/Akt activation [23] and prevents isoproterenol-induced acute injury by modulating Bcl-2/Bax balance and inactivating caspase-3 [24]. These actions align DHQ with a class of flavonoids that concurrently regulate oxidative stress, mitochondrial dysfunction, apoptosis, and ferroptosis [25,26,27]. Notably, several flavonoids have been shown to concurrently suppress apoptosis and ferroptosis in cardiovascular injury models, underscoring their therapeutic potential as multi-target agents [28,29,30]. Within this framework, DHQ represents a particularly promising candidate for mitigating chemotherapy-induced cardiotoxicity through coordinated modulation of cell death pathways.

To date, studies have established that ATO exerts its cardiotoxic effects primarily by targeting cardiomyocytes and vascular endothelial cells, resulting in myocardial damage and vascular injury [31,32]. However, the interplay between apoptotic and ferroptotic signaling pathways in this context, along with the potential role and mechanism of DHQ in modulating these processes, remains unclear. Therefore, we designed this study to systematically examine this crosstalk and to elucidate the protective function of DHQ. This investigation dissects the mechanisms underlying DHQ’s cardioprotective effects against ATO toxicity, with particular emphasis on its modulation of the AMPK/SIRT1/PGC-1α axis and the apoptosis–ferroptosis crosstalk. Our findings establish a novel therapeutic paradigm for optimizing ATO-based chemotherapy regimens through targeted cell death pathway interception.

## 2. Results

### 2.1. DHQ Alleviates ATO-Induced Cytotoxicity

To evaluate the cytotoxic effects of arsenic trioxide (ATO) and the potential protective role of dihydroquercetin (DHQ), we first performed cell viability assays. Using CCK-8 colorimetric analysis, we established distinct dose–response profiles for ATO in both AC16 human cardiomyocytes and HUVECs. The calculated IC50 values were 5.19 μM for AC16 cells and 8.26 μM for HUVECs (Figure 1a,d). Based on these results, subsequent experiments utilized ATO concentrations of 5 μM and 8 μM for AC16 cells and HUVECs, respectively. The potential of DHQ to mitigate this ATO-induced cytotoxicity was then investigated. DHQ co-treatment exhibited dose-dependent cytoprotective effects. While 50 μM DHQ showed negligible protection in AC16 cells, it significantly restored viability in HUVECs (Figure 1b,e). Notably, 100 μM DHQ provided protection in both cell types, enhancing viability without intrinsic cytotoxicity (Figure 1c,f). Complementing the viability data, morphological assessments were conducted via bright-field microscopy. Visual and morphometric analyses revealed that ATO exposure induced characteristic pathological alterations in both cell types, including cell shrinkage, rounding, and reduced density. Co-administration of 100 μM DHQ significantly attenuated these structural anomalies, preserving a healthier, more confluent cellular morphology (Figure 1g,h). Collectively, these findings demonstrate that DHQ effectively alleviates ATO-induced cytotoxicity and morphological damage in both cardiac and vascular endothelial cells.

### 2.2. DHQ Attenuates ATO-Induced Cell Apoptosis

We first examined apoptotic signaling in response to ATO exposure. In both AC16 cardiomyocytes and HUVECs, ATO downregulated Bcl-2 expression while upregulating Bax and cleaved Caspase-3 levels. These changes were effectively reversed by DHQ co-treatment (Figure 2a–d and Figure S1a–d). TUNEL staining further confirmed ATO-induced apoptosis, with increased DNA fragmentation in both cell types, whereas DHQ co-treatment reduced the proportion of TUNEL-positive cells (Figure 2g,h and Figure S1g,h). Consistent with these findings, JC-1 fluorescent analysis indicated that ATO induced mitochondrial depolarization, as evidenced by significantly decreased red-to-green fluorescence ratios. DHQ co-treatment partially restored the mitochondrial membrane potential (Figure 2e,f and Figure S1e,f). To evaluate DNA damage associated with mitochondrial apoptosis initiation, γ-H2AX immunofluorescence was performed. ATO treatment significantly increased γ-H2AX expression, an effect that was attenuated by DHQ co-administration, supporting the genoprotective role of DHQ (Figure 2i,j and Figure S1i,j). These results indicate that DHQ protects against ATO-induced cardiotoxicity and endothelial injury by inhibiting key apoptotic pathways.

### 2.3. DHQ Mitigates ATO-Induced Ferroptosis

Mitochondria, being iron-enriched organelles and primary sites of reactive oxygen species (ROS) generation, represent critical hubs in the ferroptotic cascade. In the present study, we assessed whether ATO-induced cardiotoxicity involves ferroptosis and whether DHQ can modulate this process. Western blot analysis revealed ATO downregulated the key ferroptosis regulators GPX4 and xCT in both AC16 cells and HUVECs, while DHQ co-treatment effectively restored their expression (Figure 3a–c and Figure S2a–c). Consistent with ferroptotic injury, ATO exposure increased lipid peroxidation (MDA), elevated ROS levels, and reduced antioxidant capacity (GSH and SOD). These changes were significantly reversed by DHQ intervention (Figure 3d–h and Figure S2d–h).

Furthermore, ATO triggered intracellular iron accumulation, a hallmark of ferroptosis, which was markedly attenuated by DHQ co-treatment (Figure 3i,j and Figure S2i,j). Nuclear staining with Hoechst33258 revealed ATO-induced chromatin condensation and nuclear fragmentation, whereas MitoSOX-based fluorescence quantification confirmed that DHQ reduced mitochondrial superoxide overproduction (Figure 3k,l and Figure S2k,l). Together, these data demonstrate that ATO promotes ferroptotic injury through iron overload, oxidative stress, and suppression of GPX4/xCT, and that DHQ exerts cardioprotection by restoring redox balance and inhibiting ferroptotic signaling.

### 2.4. Potential Targets of DHQ for Attenuating Cardiotoxicity

The chemical structure and SMILES notation of DHQ were obtained from PubChem (Figure 4a). A total of 232 putative DHQ targets were identified using SwissTargetPrediction, TargetNet, Super-PRED, and SEA. Cardiotoxicity-related genes (n = 35,943) and ATO targets (n = 400) were collected from CTD and GeneCards. The overlap between DHQ targets and cardiotoxicity genes yielded 222 common targets. PPI network analysis using STRING identified ten hub targets, including NFKB1, HIF1A, SRC, ESR1, BCL2, MAPK1, PPARG, STAT1, PTGS2, and MMP9 (Figure 4b). The intersection of ATO targets and cardiotoxicity genes resulted in 400 common targets, with hub targets such as TP53, AKT1, TNF, JUN, and SRC (Figure 4c).

The intersection of DHQ targets, ATO targets, and cardiotoxicity genes yielded 54 common targets (Figure 4d). PPI analysis identified hub genes including NFKB1, HIF1A, SRC, ESR1, BCL2, PPARG, PTGS2, and MMP9 (Figure 4e). GO and KEGG analyses revealed enrichment in apoptosis, ferroptosis, fatty acid metabolism, and AMPK signaling (Figure 4f–h). An integrated network positioned AMPK as a central node linking DHQ with ATO-induced cardiotoxicity (Figure 4i). Collectively, these findings suggest that DHQ exerts cardioprotective effects through multi-target regulation of apoptotic, oxidative, and metabolic pathways, with AMPK as a key hub.

### 2.5. DHQ Ameliorates ATO-Induced Apoptosis via Activating AMPK Signaling

Based on our network pharmacology predictions, we next sought to experimentally examine the role of the AMPK/SIRT1/PGC-1α axis in DHQ-mediated protection against ATO-induced cardiotoxicity. Western blot analysis confirmed that ATO exposure suppressed the activation of this pathway, as evidenced by reduced levels of phosphorylated AMPK (p-AMPK) and its downstream effectors SIRT1 and PGC-1α in both AC16 cells and HUVECs. Co-treatment with DHQ effectively restored their expression (Figure 5a–d and Figure S3a–d), suggesting DHQ may exert its protective effect through this signaling axis.

To functionally establish this dependency, we employed the AMPK inhibitor Compound C (CC). The activation of AMPK/SIRT1/PGC-1α induced by DHQ was completely abolished by CC co-treatment, returning protein levels to those observed with ATO alone (Figure 5e–h and Figure S3e–h). Furthermore, the protective effects of DHQ on apoptosis and oxidative stress were shown to be mediated through this pathway. DHQ co-treatment reversed ATO-induced alterations in the expression of apoptotic markers (Bcl-2, Bax, and cleaved Caspase-3), and this rescue was blocked by AMPK inhibition with CC (Figure 5i–k and Figure S3i–k). Similarly, the antioxidant effect of DHQ, reflected by its reduction in ATO-induced ROS accumulation, was also negated by CC (Figure 5l,m and Figure S3l,m). In summary, these functional validation experiments demonstrate that the activation of the AMPK/SIRT1/PGC-1α signaling axis plays a critical role in DHQ to attenuate ATO-induced cardiomyocyte and endothelial cell injury, primarily by inhibiting apoptosis and mitigating oxidative stress.

### 2.6. DHQ Ameliorates ATO-Induced Ferroptosis via Activating AMPK Signaling

To further explore whether DHQ’s protection involves ferroptosis regulation via the AMPK/SIRT1/PGC-1α axis, we examined key ferroptotic markers under pathway modulation. Western blot analysis demonstrated that DHQ co-treatment (100 μM) markedly upregulated the expression of ferroptosis-defense proteins GPX4 and xCT in both AC16 cells and HUVECs exposed to ATO. In parallel, FerroOrange fluorescence staining showed a pronounced decrease in intracellular labile iron, a hallmark of ferroptotic stress. Critically, these protective alterations—enhanced GPX4/xCT expression and reduced iron accumulation—were completely abrogated by co-administration of the AMPK inhibitor Compound C (CC), confirming that DHQ’s anti-ferroptotic effects depend on AMPK activation (Figure 6a–e and Figure S4a–e). We next assessed mitochondrial oxidative stress, a key driver of ferroptosis. MitoSOX Red staining revealed that DHQ effectively attenuated ATO-induced mitochondrial superoxide overproduction. Notably, this antioxidant effect was also abolished when AMPK was inhibited by CC, indicating that DHQ’s ability to scavenge mitochondrial ROS is likewise mediated through the AMPK/SIRT1/PGC-1α pathway (Figure 6f,g and Figure S4f,g).

Furthermore, we successfully inhibited the expression of AMPK in the primary cardiomyocytes of newborn mice using the siRNA technique (Figure S5a), further verifying this mechanism. The experimental results showed that under the ATO condition, siAMPK could inhibit the activation effect of DHQ on the AMPK pathway, and restore the expression of AMPK pathway, apoptosis and ferroptosis-related proteins to the level as in the case of ATO used alone (Figure S5b–h). The ROS detection results indicated that under the ATO condition, compared with the DHQ group, the ROS and mitochondrial ROS in the cells of the DHQ and siAMPK co-incubation group increased (Figure S5i–l).

Together, these findings demonstrate that activation of the AMPK/SIRT1/PGC-1α signaling axis contributes substantially to DHQ’s protection against ATO-induced ferroptosis. The mechanism involves the coordinated restoration of GPX4/xCT-dependent anti-lipid peroxidation defenses, the reduction in cytotoxic iron overload, and the alleviation of mitochondrial oxidative stress, thereby preserving cardiomyocyte and endothelial viability under arsenic insult.

## 3. Discussion

The clinical success of ATO in treating APL is counterbalanced by its well-documented cardiotoxicity, which constrains therapeutic efficacy and patient safety [9]. This necessitates the discovery of effective cardioprotective adjuvants. Through integrated network pharmacology and experimental validation, the present study identifies DHQ as a novel cardioprotective compound against ATO-induced myocardial injury. DHQ coordinately modulates three pathological processes: oxidative stress, apoptotic signaling, and ferroptosis cascades. Mechanistic investigations establish that DHQ exerts its therapeutic effects by activating the AMPK/SIRT1/PGC-1α signaling axis. The essential nature of this pathway was supported, as pharmacological inhibition of AMPK with CC and siRNA abrogated DHQ’s ability to mitigate mitochondrial dysfunction, oxidative damage, and both forms of regulated cell death.

Contemporary management of ATO-induced cardiotoxicity faces significant challenges, largely attributable to incomplete comprehension of cardiac cellular dynamics [33]. Although cardiomyocytes (CMs) comprise 20–33% of myocardial cellularity, emerging evidence underscores the pathophysiological contributions of non-myocyte populations—particularly endothelial cells (ECs), cardiac fibroblasts, and resident immune cells—in maintaining cardiac homeostasis and mediating injury responses [34,35,36]. Our findings contribute to this evolving paradigm. Our comparative cytotoxicity profiling demonstrated conserved vulnerability between AC16 cells (IC_50_ = 5.19 μM) and HUVECs (IC_50_ = 8.26 μM) following 24 h ATO exposure. Multi-modal assessment incorporating ROS flux analysis (DCFDA probe), DNA damage quantification (γ-H2AX foci enumeration), apoptotic index determination (TUNEL assay), and ferroptosis progression monitoring (FerroOrange iron detection) revealed conserved toxicological mechanisms across cellular subtypes. This observation challenges the prevailing cardiomyocentric model of ATO cardiotoxicity, establishing the endothelial compartment as a critical mediator in arsenic-induced myocardial pathogenesis.

Mechanistically, our experimental data establish the AMPK/SIRT1/PGC-1α axis as a critical mediator of DHQ-mediated cytoprotection. While network pharmacology also identified other hub targets (e.g., NFKB1, HIF1A, PPARG, PTGS2), AMPK was prioritized for validation based on three considerations: its established role as a master regulator of energy metabolism and mitochondrial function [37,38], its integration of oxidative stress and cell death signaling [39], and its significant enrichment in KEGG pathway analysis. Nevertheless, DHQ’s protective effects likely involve coordinated regulation of multiple pathways rather than a single axis—NFKB1 in inflammation [40], PPARG in lipid metabolism and ferroptosis [41,42], and PTGS2 in oxidative stress responses [43]—which may converge to amplify the overall benefit. At the molecular level, AMPK activation enhances SIRT1 activity, which deacetylates and activates PGC-1α, promoting antioxidant gene expression and mitochondrial quality control [37,44,45]. Importantly, activated SIRT1 can upregulate xCT to boost cystine uptake and glutathione synthesis, strengthening GPX4-mediated defense against ferroptosis [46]. Simultaneously, this axis inhibits pro-apoptotic signaling [47]. Our data show that DHQ significantly restored mitochondrial membrane potential and reduced mitochondrial ROS overproduction. Thus, via AMPK/SIRT1/PGC-1α activation, DHQ protects mitochondrial integrity from oxidative insult, thereby alleviating both apoptotic and ferroptotic cell death. Direct assessment of mitochondrial bioenergetic recovery using Seahorse or high-resolution respirometry is warranted in future studies. Additionally, transcriptomic or proteomic approaches may clarify the relative contributions of other hub targets.

Oxidative stress is a cardinal mechanism in ATO cardiotoxicity, causing direct myocardial injury and epigenetic dysregulation [14]. Consistent with prior toxicological findings in rodent models that sodium arsenite increased myocardial MDA levels while reducing SOD and GSH activities in rat models [10]. Our data show ATO-induced redox disequilibrium, characterized by ROS overproduction and depletion of antioxidant reserves (GSH, SOD), in both cardiomyocyte and endothelial models. DHQ, a bifunctional redox-modulating agent with established DNA repair-enhancing properties [48], effectively intercepted this oxidative cascade. Its efficacy manifested not only as direct ROS scavenging but also as a significant alleviation of the downstream DNA damage response, highlighting its capacity to preserve genomic integrity—a critical aspect often compromised in chemotherapy-associated toxicity.

Our investigation delineates a dual cell death landscape activated by ATO, with concurrent engagement of apoptosis (Bcl-2/Bax dysregulation, caspase-3 cleavage, DNA fragmentation) and ferroptosis (GPX4/xCT suppression, iron accumulation, lipid peroxidation) in both cell types. We propose a model wherein ATO-induced oxidative stress serves as a common upstream trigger. It provokes mitochondrial dysfunction—loss of membrane potential and mROS overproduction—which can both initiate intrinsic apoptosis and, by exacerbating lipid peroxidation and depleting GPX4/xCT-dependent defenses, promote ferroptosis [49,50]. Rather than invoking direct pathway crosstalk, we suggest that their convergence occurs at the level of mitochondrial oxidative injury, which amplifies cellular damage. This aligns with emerging evidence that apoptotic and ferroptotic pathways exhibit redox-driven synergy in cardiovascular disorders, forming a ROS-mediated feedforward loop: primary oxidative insult triggers both death cascades, glutathione depletion aggravates iron dyshomeostasis, and mitochondrial permeability transition further amplifies oxidative stress [50,51,52]. This intertwined model explains the enhanced efficacy of DHQ, which, via AMPK/SIRT1/PGC-1α activation, interrupts the upstream oxidative cascade and coordinately alleviates both death programs.

Our findings position DHQ within the broader framework of flavonoid pharmacology. Structurally related flavonoids—quercetin, luteolin, and acacetin—act as pleiotropic regulators of redox homeostasis, mitochondrial integrity, and cell death pathways [25,26,27]. Quercetin, differing only by a C2–C3 double bond, exhibits antioxidant, vasodilatory, and antiplatelet effects [53]. However, DHQ is more potent against xanthine oxidase-derived superoxide, while quercetin uniquely inhibits ADP-induced platelet aggregation, highlighting distinct profiles [54]. Luteolin protects against ischemia/reperfusion injury by inhibiting both apoptosis and ferroptosis through mitochondrial protection [29]. Acacetin ameliorates cardiac hypertrophy via Sirt1-mediated AMPK/PGC-1α activation, strikingly similar to DHQ [55]. This convergence suggests AMPK/SIRT1/PGC-1α activation as a shared mechanistic signature of cardioprotective flavonoids. Future studies should systematically delineate structure-activity relationships that distinguish DHQ from its analogs.

Beyond mechanism, our findings hold translational relevance. Flavonoid-based adjunct therapies are gaining attention in oncology for their multi-target cytoprotective and antioxidative properties [56]. Flavonoids such as quercetin, luteolin, and isorhamnetin protect normal cells from chemotherapy-induced damage by modulating redox balance, mitochondrial function, and cell death pathways [57]. Emerging formulation strategies—nanoparticles, cyclodextrin systems, and albumin-based nanocarriers—offer promise in overcoming the poor bioavailability that has limited clinical translation [58,59]. These innovations, together with our findings, support future exploration of DHQ as a clinically viable cardioprotective adjuvant in ATO-based regimens.

A critical translational concern is whether DHQ-mediated cytoprotection could inadvertently protect malignant cells from ATO. Theoretically, antioxidant or anti-apoptotic interventions risk shielding cancer cells [60,61,62]. However, several considerations suggest differential effects. Cancer cells, including APL cells, exhibit metabolic reprogramming (heightened oxidative stress and aerobic glycolysis) that may render them less reliant on AMPK-mediated survival pathways compared to normal cardiomyocytes and endothelial cells [63,64]. Certain flavonoids can selectively protect normal cells while sensitizing cancer cells by modulating redox balance and apoptotic thresholds [65,66]. Moreover, the AMPK/SIRT1/PGC-1α axis, while protective in normal cells, may suppress proliferation or enhance chemosensitivity in cancer cells under specific conditions [67,68,69]. Nevertheless, these considerations require direct experimental verification. We acknowledge the lack of data on DHQ’s impact on ATO’s anti-leukemic efficacy as a significant limitation. Future in vivo studies using APL xenograft or orthotopic models are necessary to determine whether DHQ preserves or enhances ATO’s therapeutic index. Until such evidence is available, any claim of clinical applicability would be premature.

## 4. Materials and Methods

### 4.1. Prediction of DHQ-Related Targets

The two-dimensional (2D) chemical structure and canonical SMILES notation of DHQ were acquired through querying the PubChem database (https://pubchem.ncbi.nlm.nih.gov/, accessed on 27 July 2026) using “Dihydroquercetin” and “taxifolin” as the search term. The target genes of Dihydroquercetin were obtained by searching the SwissTargetPrediction (http://www.swisstargetprediction.ch/, accessed on 27 July 2026), TargetNet (http://targetnet.scbdd.com/calcnet/index/, accessed on 27 July 2026), Super-PRED (https://prediction.charite.de/index.php?site=chemdoodle_search_target, accessed on 27 July 2026) and SEA (https://sea.bkslab.org/, accessed on 27 July 2026) databases. The duplicate items were discarded and the key targets were screened out, thus obtaining the target gene names corresponding to Dihydroquercetin.

### 4.2. Prediction of Gene Targets for ATO

By searching for the targets of Arsenic Trioxide through the GeneCards (https://www.genecards.org/) and CTD (https://ctdbase.org/) databases, duplicate items were eliminated, and the key targets were selected. Thus, the names of the target genes corresponding to Arsenic Trioxide were obtained.

### 4.3. Prediction of Gene Targets for Cardiotoxicity

The screening of genes related to cardiac toxicity is based on GeneCards (https://www.genecards.org/) and CTD (https://ctdbase.org/). By inputting the keyword “Cardiotoxicity”, relevant target genes for cardiac toxicity are retrieved and collected. After summarizing the results and deleting duplicate genes, the cardiac toxicity target genes are obtained. The intersection of the target genes for Arsenic Trioxide and cardiac toxicity, and the target genes for Dihydroquercetin and cardiac toxicity were calculated. The intersection of these two sets of target genes represents the potential key target points for Arsenic Trioxide and Dihydroquercetin in influencing cardiac toxicity.

### 4.4. PPI Network

Protein–protein interaction (PPI) networks for the intersecting targets were constructed via the STRING database (https://string-db.org/; Homo sapiens taxonomy). Interaction data were retrieved in TSV format and imported into Cytoscape 3.9.1 for network visualization and topological evaluation. Core nodes were identified through the evaluation of critical topological metrics, including degree centrality, betweenness centrality, and closeness centrality.

### 4.5. GO and KEGG Enrichment Analysis

Gene Ontology (GO) functional annotation and Kyoto Encyclopedia of Genes and Genomes (KEGG) pathway enrichment analyses were conducted utilizing the Metascape resource (http://metascape.org/) to elucidate the biological functions and signaling cascades associated with hub targets. Results were graphically represented through clustered bar plots and annotated bubble charts.

### 4.6. Chemicals and Reagents

ATO raw liquid (1 mg/mL) was provided by Harbin YI-DA Pharmaceutical Co., Ltd. (Harbin, China). DHQ (HY-N0136, with a purity of 99.97 %), Compound C (HY-13418A), and MitoSOX assay were obtained from MedChemExpress (MCE) (Monmouth Junction, NJ, USA). Malondialdehyde (MDA), glutathione (GSH), and superoxide dismutase (SOD) assay kits were purchased from Jiancheng Bioengineering Institute (Nanjing, China). ROS fluorescence detection kits, TUNEL staining kits, Hoechst 33258 staining, cell counting Kit-8, and mitochondrial membrane potential assay kits with JC-1 were purchased from Beyotime (Shanghai, China). AC16 cells and HUVECs were provided by the Myocardial Ischemia Laboratory at Harbin Medical University.

### 4.7. Cell Culture

AC16 cells and HUVECs were purchased from Procell LifeScience & Technology Co., Ltd. AC16 cells and HUVECs were cultured in high-glucose Dulbecco’s Modified Eagle Medium (DMEM) supplemented with 10% fetal bovine serum and 1% penicillin/streptomycin. The cells were cultured in an incubator (37 °C, 5% CO_2_). Passage numbers represent cells used at passages 3–6, and culture confluence experiments were performed at 70–80% confluence. The AC16 cells were divided into 4 groups: (a) control group: treated with culture medium for 24 h; (b) DHQ group: treated with 100 µM DHQ for 24 h; (c) ATO group: treated with 5 µM ATO for 24 h; (d) ATO + DHQ group: pre-treated with 100 µM DHQ for 12 h followed by incubation with 5 µM ATO for 24 h. The HUVECs were divided into 4 groups: (e) control group: treated with culture medium for 24 h; (f) DHQ group: treated with 100 µM DHQ for 24 h; (g) ATO group: treated with 8 µM ATO for 24 h; (h) ATO + DHQ group: pre-treated with 100 µM DHQ for 12 h followed by incubation with 8 µM ATO for 24 h.

### 4.8. Cell Viability Assay

After drug treatment, 10 μL CCK-8 was added to each well and thoroughly mixed, and the cells remained at 37 °C for 1 h. Meanwhile, to avoid interference, bubble formation was prevented. The absorbance of the samples was measured at a wavelength of 450 nm using a microplate reader (Molecular Devices, San Jose, CA, USA).

### 4.9. Bright-Field and Immunofluorescent Imaging

Bright-field and fluorescence microscopy were used to investigate the effects of ATO and DHQ on the morphology of AC16 and HUVEC cells. The cells were fixed in 4% paraformaldehyde for 10 min, permeabilized with 0.1% Triton X-100 for 10 min, and sealed with 0.1% goat serum for 1 h. Cells were incubated first with primary antibody γ-H2AX (1:200, A11097, ABclonal, Woburn, MA, USA) at 4 °C overnight and then with fluorescein isothiocyanate (FITC)-labeled goat anti-rabbit IgG antibody (1:200, A0468, Beyotime, Shanghai, China) for 1 h. The cells were incubated with DAPI (Beyotime, Shanghai, China). Images were acquired using a fluorescence microscope (Zeiss, Jena, Germany).

### 4.10. Determination of Oxidative Stress and ROS

Intracellular levels of MDA, GSH, and SOD were determined using assay kits and spectrophotometry. After drug treatment, cells were washed with PBS and then incubated with 10 µM DCFH-DA at 37 °C for 30 min in the dark, followed by observation under a fluorescence microscope.

### 4.11. TUNEL Assay

The TUNEL assay kit was used to label fragmented DNA to detect cell death. DNA fragments could be specifically stained by TUNEL, producing green fluorescence, while DAPI could label intact and fragmented DNA, resulting in blue fluorescence. Following drug treatment, cells were washed twice with PBS, incubated with 100 µL of TUNEL detection solution for 1 h, washed three times with PBS, incubated with DAPI for 5 min, and observed under a fluorescence microscope.

### 4.12. Determination of the MMP

Following drug treatment, the cells were incubated with JC-1 working solution at 37 °C for 30 min in the dark. Following two washes with JC-1 buffer, the cells were observed under a fluorescence microscope. A decrease in the red/green fluorescence intensity ratio indicated a loss of mitochondrial membrane potential (MMP).

### 4.13. Western Blot Analysis

Protein separation was achieved using 10% SDS-PAGE, followed by transfer to a PVDF membrane. The membrane was then incubated with primary antibodies: Bax (1:1000, abways), Bcl-2 (1:1000, abways), Caspase-3 (1:1000, abways), GPX4 (1:1000, abways), xCT (1:1000, abways), AMPK (1:1000, Immunoway), P-AMPK (1:1000, MCE), SIRT1 (1:1000, MCE), PGC-1α (1:1000, Wanleibio), and β-actin (1:2000, Affinity). After three washes with PBST, the membrane was incubated with secondary antibodies (1:2000, Invitrogen, Waltham, MA, USA) for 1 h. Subsequently, the PVDF membrane was scanned using a Bio-Rad gel imaging system (Hercules, CA, USA), and ImageJ software (version 1.54) was used to quantify the protein bands.

### 4.14. Determination of the Intracellular Iron

Intracellular iron concentrations were determined using FerroOrange (Dojindo, Kumamoto, Japan). Cells were washed three times with serum-free medium and then incubated with 1 µM FerroOrange in serum-free medium for 30 min at 37 °C. Images were acquired using a fluorescence microscope.

### 4.15. MitoSOX Assay

The cells were washed twice with serum-free medium and then diluted with serum-free cell culture medium to prepare a 1 μM working solution, which was maintained at room temperature for 5–30 min in the dark. Subsequently, the cells were washed twice with culture medium for 5 min each time. Images were acquired using a fluorescence microscope.

### 4.16. Hoechst 33258 Staining

Following drug treatment, the culture medium was aspirated, and the cells were fixed with 0.5 mL of 4% paraformaldehyde for 10 min. The cells were then washed twice with PBS for 3 min each time. Next, the cells were incubated with 0.5 mL of Hoechst 33258 staining solution in the dark for 5 min. After incubation, the cells were washed twice with PBS for 3 min each time.

### 4.17. Statistical Analysis

The data were presented as the mean ± standard deviation (SD). The data were plotted using GraphPad Prism software version 9.0 (GraphPad Software, Inc., La Jolla, CA, USA). Statistical analysis was performed using IBM SPSS Statistics software (version 32.0). Unpaired *t*-tests were used for comparisons between two groups, and one-way analysis of variance (ANOVA) was used for comparisons among multiple groups. *p* < 0.05 was considered statistically significant.

## 5. Conclusions

This investigation systematically delineated the molecular targets and cardioprotective mechanisms of DHQ through integrated network pharmacology-based approaches and experimental validation. Mechanistically, we demonstrated that DHQ-mediated cardioprotection against ATO-induced cardiac injury involves concerted inhibition of apoptosis signaling, ferroptosis, and oxidative stress cascades, primarily via activation of the AMPK/SIRT1/PGC-1α signaling axis. These findings establish a novel therapeutic framework for mitigating chemotherapy-induced cardiotoxicity, with particular clinical relevance for ATO-based regimens. Furthermore, our work provides two significant conceptual advances. First, by employing a dual cardiomyocyte–endothelial cell model, we underscore the critical vulnerability of the cardiac endothelium to ATO, arguing for a more holistic “cardiovascular” rather than purely “myocardial” perspective on its toxicity. Second, we elucidate the pathogenic interplay between apoptosis and ferroptosis in this context, demonstrating how their redox-driven synergy exacerbates injury and highlighting the therapeutic advantage of agents capable of disrupting this crosstalk. While these in vitro findings are robust and promising, they form an essential foundation for subsequent research. Future work must include validation in preclinical in vivo models of ATO cardiotoxicity, detailed pharmacokinetic and safety studies to assess DHQ’s suitability as an adjuvant, and exploration of its potential to allow for safer dose escalation of ATO. Collectively, this research positions DHQ as a compelling candidate for development as a multi-target cardioprotectant in oncology supportive care.

## Figures and Tables

**Figure 1:**
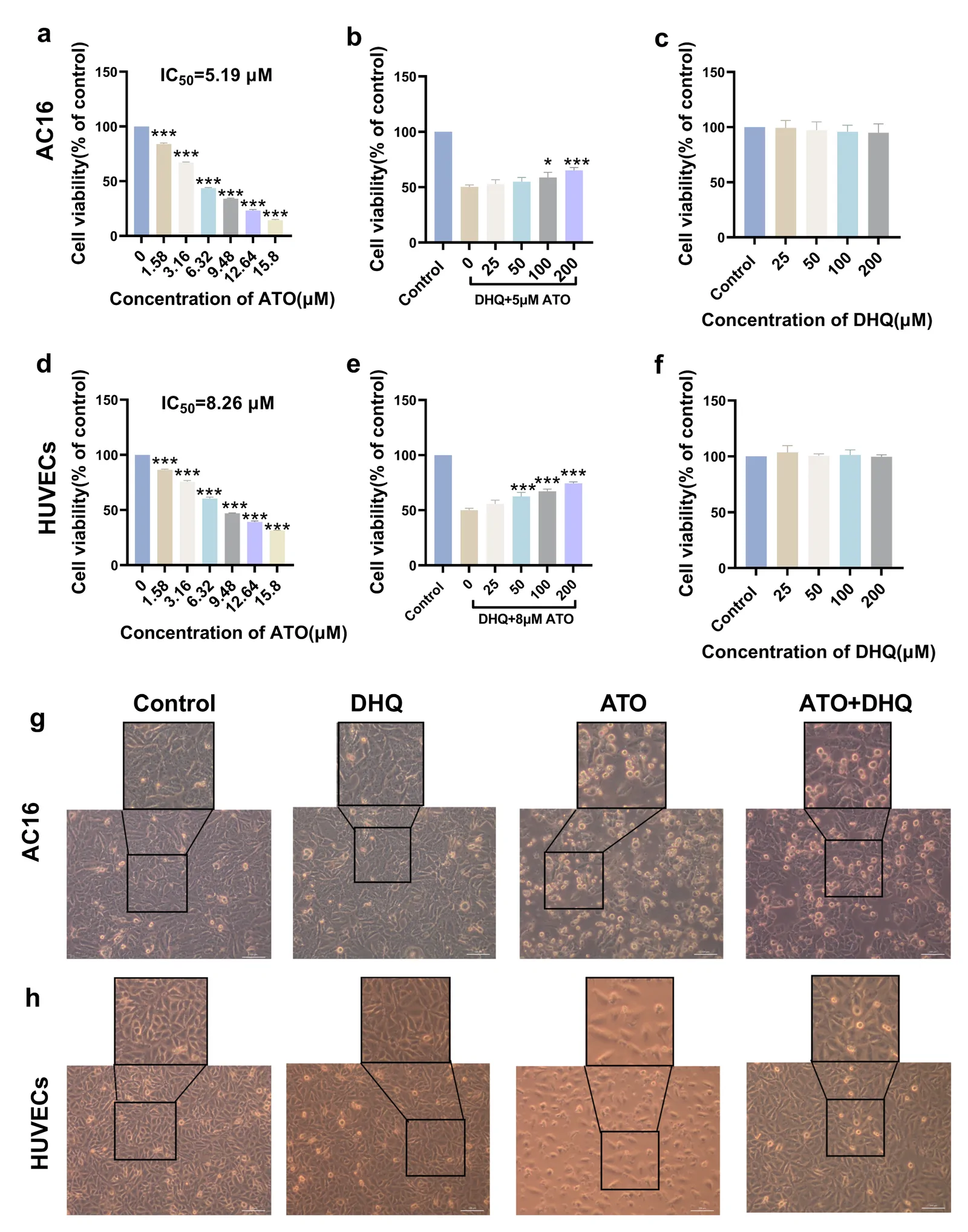
The effects of ATO and DHQ on the viability and morphology of AC16 cells and HUVECs. ATO induced a decrease in the viability of AC16 cells (**a**) and HUVECs (**d**). DHQ mitigated the effect of ATO on the viability of AC16 cells (**b**) and HUVECs (**e**). Treatment with DHQ alone (25–200 µM) showed no cytotoxic effects on AC16 cells (**c**) and HUVECs (**f**). Phase contrast imaging was performed to observe the morphological alteration in AC16 cells (**g**) and HUVECs (**h**). Scale bar = 100 µm (magnification: 10×). Values are mean ± SD for each group. * *p* < 0.05, *** *p* < 0.001, *n* ≥ 3.

**Figure 2:**
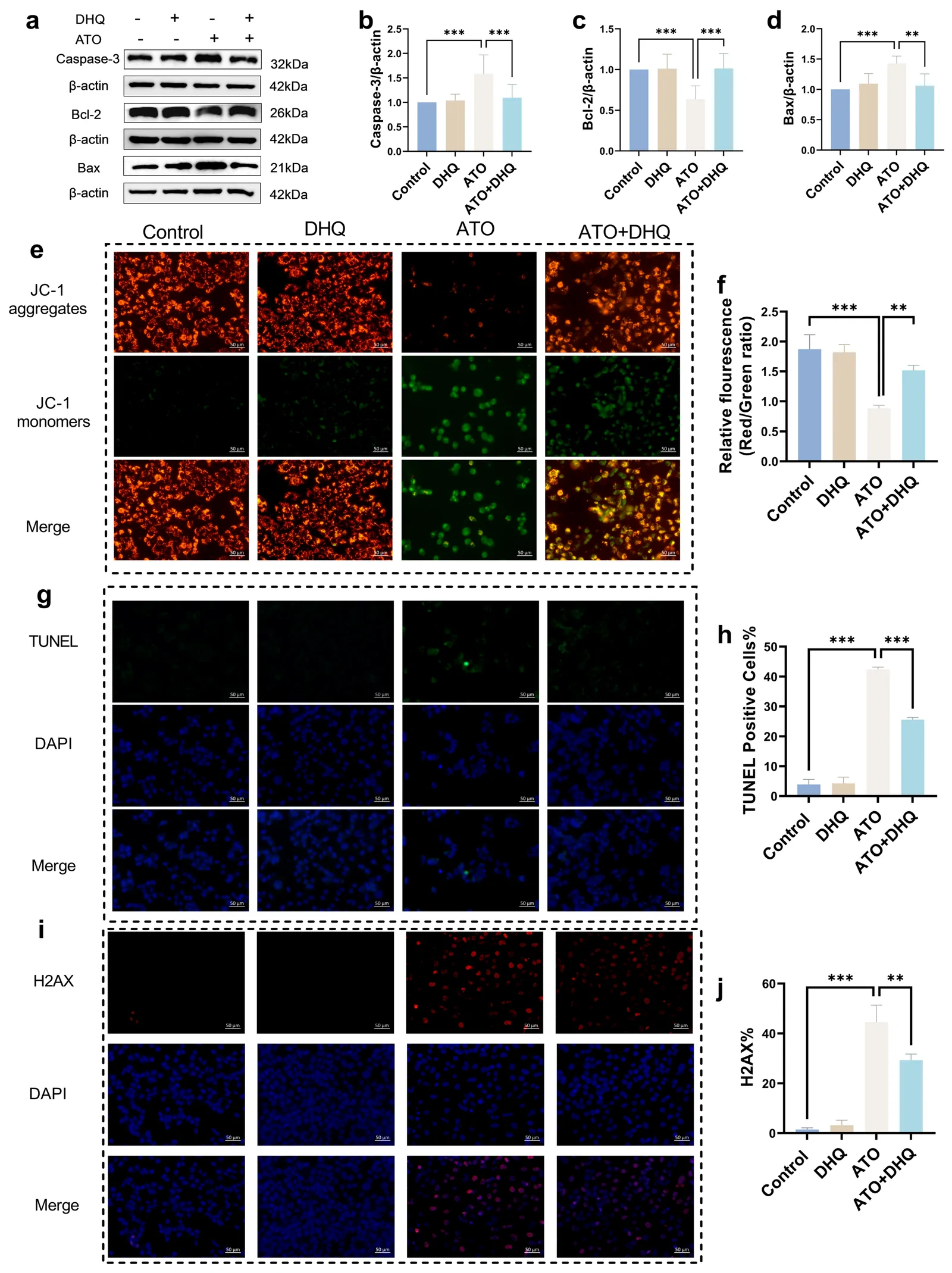
DHQ attenuates ATO-induced cell apoptosis in AC16 cells. (**a**) Western blotting analysis of Caspase-3, Bax, and Bcl-2 in AC16 cells. (**b**–**d**) Quantitative analysis of Bax/β-actin (*n* = 4), Bcl-2/β-actin (*n* = 8), and Caspase-3/β-actin (*n* = 10) in AC16 cells. (**e**) Representative fluorescence images of JC-1 staining. Scale bar = 50 µm (magnification: 20×). (**f**) Quantitative analysis of the relative fluorescence (red/green ratio) in AC16 cells. (**g**) Representative fluorescence images of TUNEL staining. Scale bar = 50 µm (magnification: 20×). (**h**) Quantitative analysis of TUNEL-positive cells in AC16 cells. (**i**) Representative fluorescence images of H2AX staining. Scale bar = 50 µm (magnification: 20×). (**j**) Quantitative analysis of the relative fluorescence (red/green ratio) in AC16 cells. Values are mean ± SD for each group. ** *p*< 0.01, *** *p* < 0.001, *n* ≥ 3.

**Figure 3:**
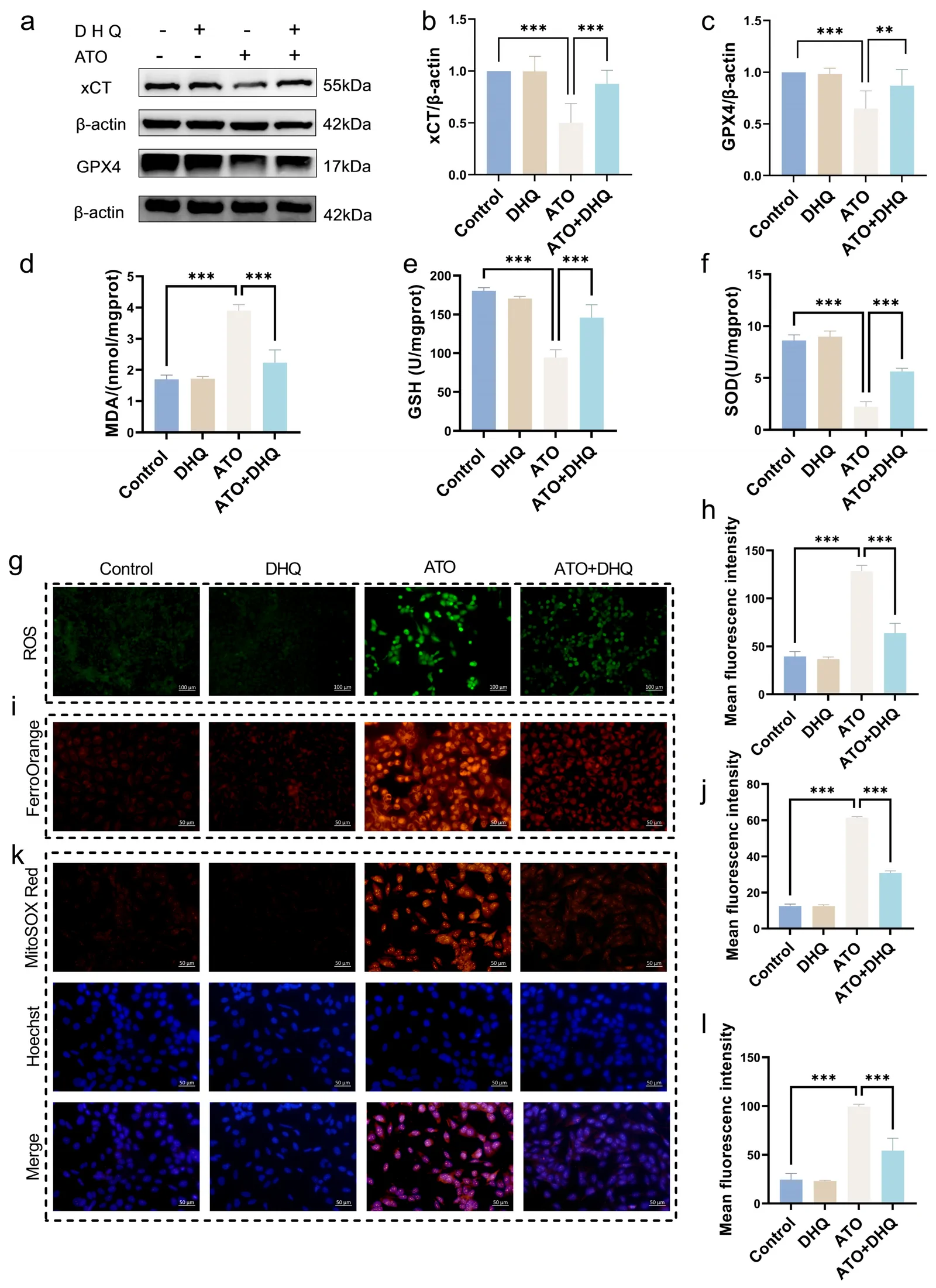
The effects of ATO and DHQ on ferroptosis in AC16 cells. (**a**) Western blotting analysis of xCT and GPX4 levels in AC16 cells. (**b**,**c**) Quantitative analysis of xCT/β-actin (*n* = 6) and GPX4/β-actin (*n* = 3) in AC16 cells. (**d**) The MDA levels in AC16 cells. (**e**) The GSH activity in AC16 cells. (**f**) The SOD activity in AC16 cells. (**g**) Representative fluorescence images of ROS staining in AC16 cells. Scale bar = 100 µm (magnification: 10×). (**h**) Quantitative analysis of ROS fluorescence intensity in AC16 cells. (**i**) Representative fluorescence images of iron ion staining in AC16 cells. Scale bar = 50 µm (magnification: 20×). (**j**) Quantification of the fluorescence intensity of iron ions in AC16 cells. (**k**) Representative fluorescence images of MitoSOX. Scale bar = 50 µm (magnification: 20×). (**l**) Quantification of the fluorescence intensity of MitoSOX in AC16 cells. Values are mean ± SD for each group. ** *p* < 0.01, *** *p* < 0.001, *n* ≥ 3.

**Figure 4:**
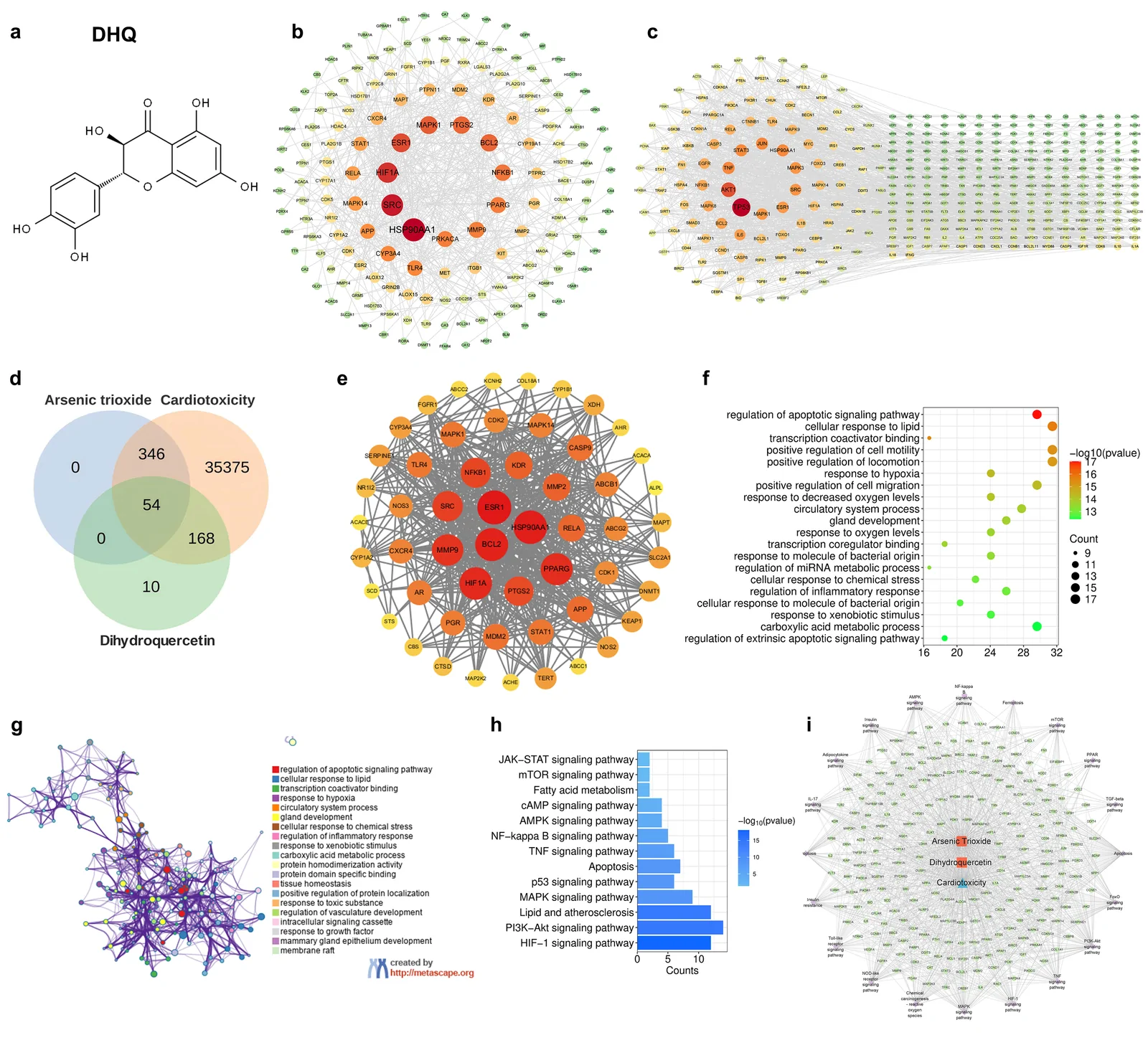
Prediction of the targets of DHQ for attenuating cardiotoxicity. (**a**) Molecular structure of DHQ. (**b**) PPI network of DHQ targets in alleviating cardiotoxicity. (**c**) PPI network of ATO targets in alleviating cardiotoxicity. (**d**) Overlapping targets between DHQ, ATO and cardiotoxicity. (**e**) PPI network of the common targets. (**f**,**g**) GO functional analysis of DHQ and ATO targets in alleviating cardiotoxicity. (**h**) KEGG pathway enrichment analysis of DHQ and ATO targets in alleviating cardiotoxicity. (**i**) Drug–disease-signaling pathway network.

**Figure 5:**
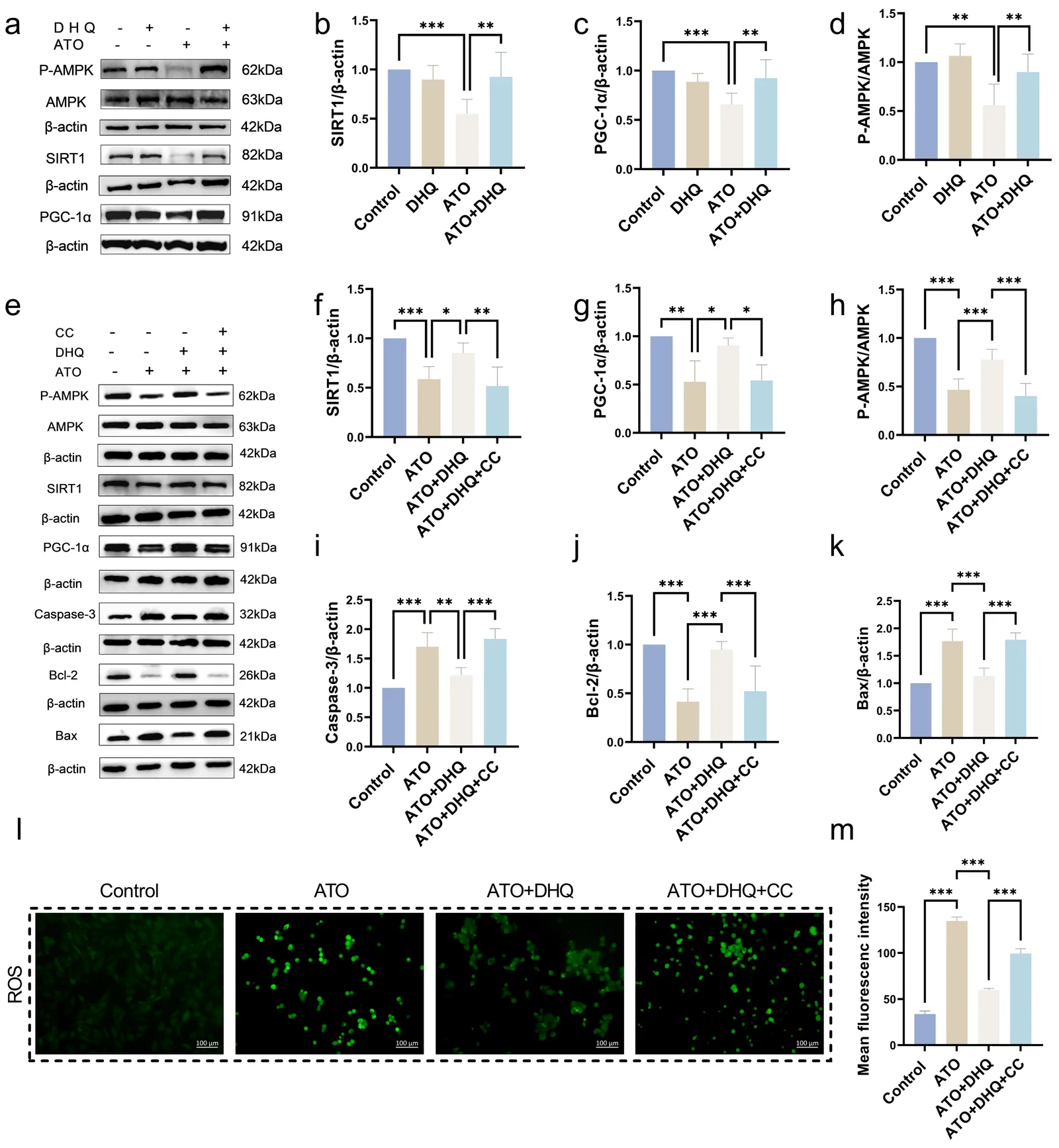
DHQ ameliorates ATO-induced apoptosis by activating the AMPK/SIRT1/PGC-1α signaling pathway in AC16 cells. (**a**) Western blotting analysis of P-AMPK, AMPK, SIRT1 and PGC-1α levels in AC16 cells. (**b**–**d**) Quantitative analysis of P-AMPK/AMPK (n = 4), SIRT1/β-actin (n = 6) and PGC-1α/β-actin (n = 6) in AC16 cells. (**e**) Western blotting analysis of P-AMPK, AMPK, SIRT1, PGC-1α, Caspase-3, Bcl-2, and Bax levels in AC16 cells. (**f**–**k**) Quantitative analysis of P-AMPK/AMPK (n = 5), SIRT1/β-actin (n = 5), PGC-1α/β-actin (n = 4), Bax/β-actin (n = 5), Bcl-2/β-actin (n = 6), and Caspase-3/β-actin (n = 5) in AC16 cells. (**l**,**m**) Representative fluorescence images of ROS staining in AC16 cells. Scale bar = 100 µm (magnification: 10×). Values are mean ± SD for each group. * *p* < 0.05, ** *p* < 0.01, *** *p* < 0.001, *n* ≥ 3.

**Figure 6:**
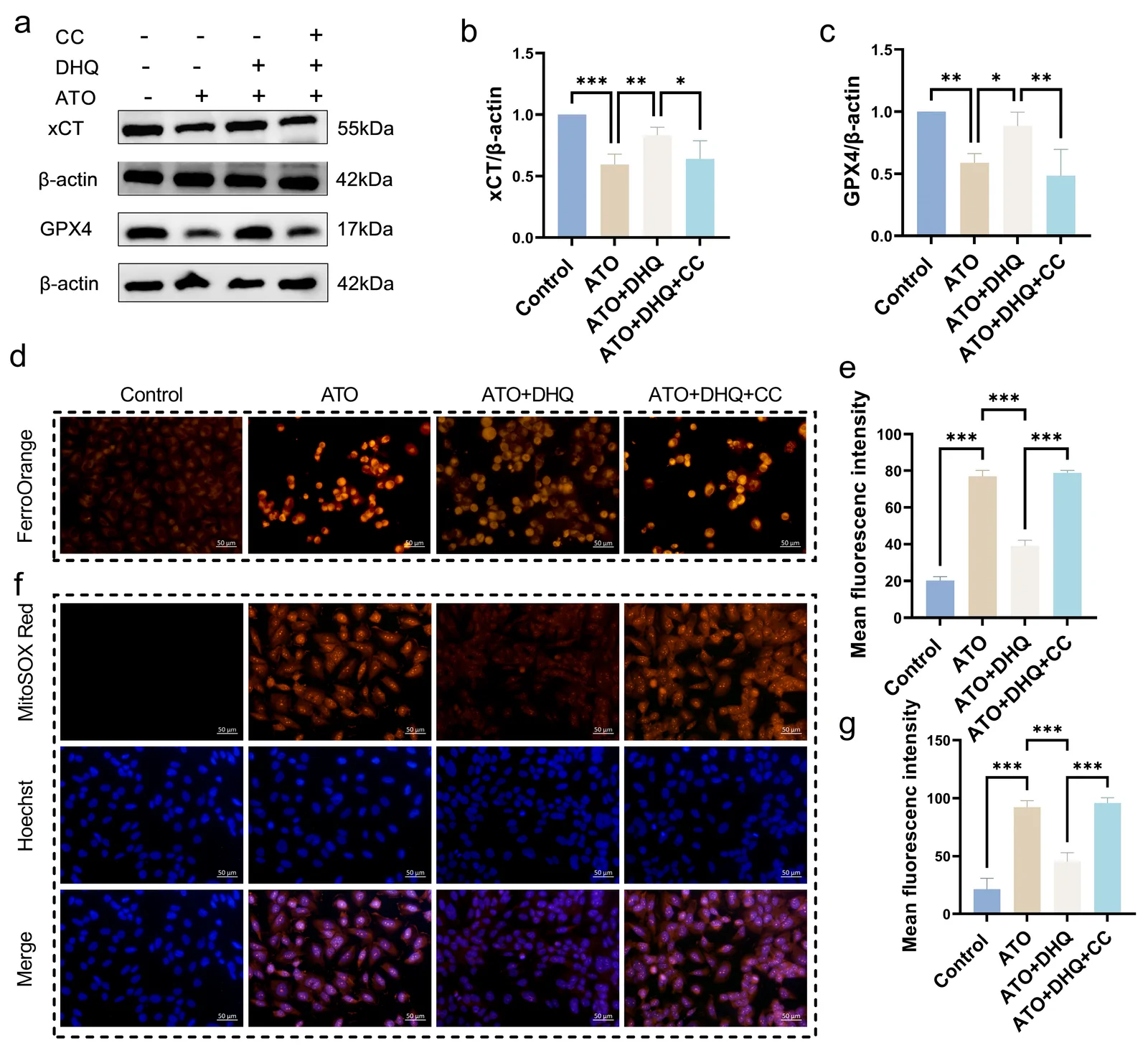
DHQ ameliorated ATO-induced ferroptosis by the AMPK/SIRT1/PGC-1α signaling pathway in AC16 cells. (**a**) Western blotting analysis of GPX4 and xCT levels in AC16 cells. (**b**,**c**) Quantitative analysis of xCT/β-actin (n = 5) and GPX4/β-actin (n = 4) in AC16 cells. (**d**) Representative fluorescence images of iron ion staining in AC16 cells. Scale bar = 50 µm (magnification: 20×). (**e**) Quantification of the fluorescence intensity of iron ions in AC16 cells. (**f**) Representative fluorescence images of MitoSOX. Scale bar = 50 µm (magnification: 20×). (**g**) Quantitative analysis of the relative fluorescence in AC16 cells. Values are mean ± SD for each group. * *p* < 0.05, ** *p* < 0.01, *** *p* < 0.001, *n* ≥ 3.

## Data Availability

The original contributions presented in this study are included in the article/Supplementary Materials. Further inquiries can be directed to the corresponding authors.

## Supplementary Materials

The following supporting information can be downloaded at: https://www.mdpi.com/article/10.3390/ijms27198753/s1.

## Abbreviations

The following abbreviations are used in this manuscript:

APL	Acute promyelocytic leukemia
ATO	Arsenic trioxide
CCK-8	Cell Counting Kit-8
DAPI	4′,6-Diamidino-2-phenylindole
DCFH-DA	2′,7′-Dichlorofluorescein diacetate
DHQ	Dihydroquercetin
DMEM	Dulbecco’s Modified Eagle’s Medium
GSH	Glutathione
MDA	Malondialdehyde
MMP	Mitochondrial membrane potential
PBS	Phosphate-buffered saline
PGC-1α	Peroxisome proliferator-activated receptor gamma coactivator 1-alpha
ROS	Reactive oxygen species
SIRT1	Sirtuin 1
SOD	Superoxide dismutase
