# Supplementary material for: Dihydroquercetin Activates AMPK/SIRT1/PGC-1α Signaling to Attenuate Arsenic Trioxide-Induced Apoptosis and Ferroptosis in AC16 Cells and HUVECs
Source: Int J Mol Sci. 2026 Sep 30;27(19):8753. doi: 10.3390/ijms27198753 (PMC13665449; doi:10.3390/ijms27198753)
Supplement: Supplementary file 1 [file ijms-27-08753-s001.zip › ijms-4390502-supplementary.pdf]

Supplementary Information for

Dihydroquercetin Activates AMPK/SIRT1/PGC-1α Signaling to Attenuate Arsenic Trioxide-Induced Apoptosis and Ferroptosis in AC16 Cells and HUVECs

Wenlei Zhang [†], Rui Duan [†], Xinsheng Duan, Sijing Tao, Haoxuan Li, Jinyan Li, Xinru Wang, Lijuan Yue, Kai Ren, Tianyu Zhao, Meihua Guo, Xiaoxiang Guan * and Xin Hai *

Department of Pharmacy, First Affiliated Hospital of Harbin Medical University, 23 YouZheng Str, Nangang District, Harbin 150007, China; 13523229801@163.com (R.D.)
*Correspondence: guanxiaoxiang@hrbmu.edu.cn (X.G.); haixin@hrbmu.edu.cn (X.H.)
[†]These authors contributed equally to this work.

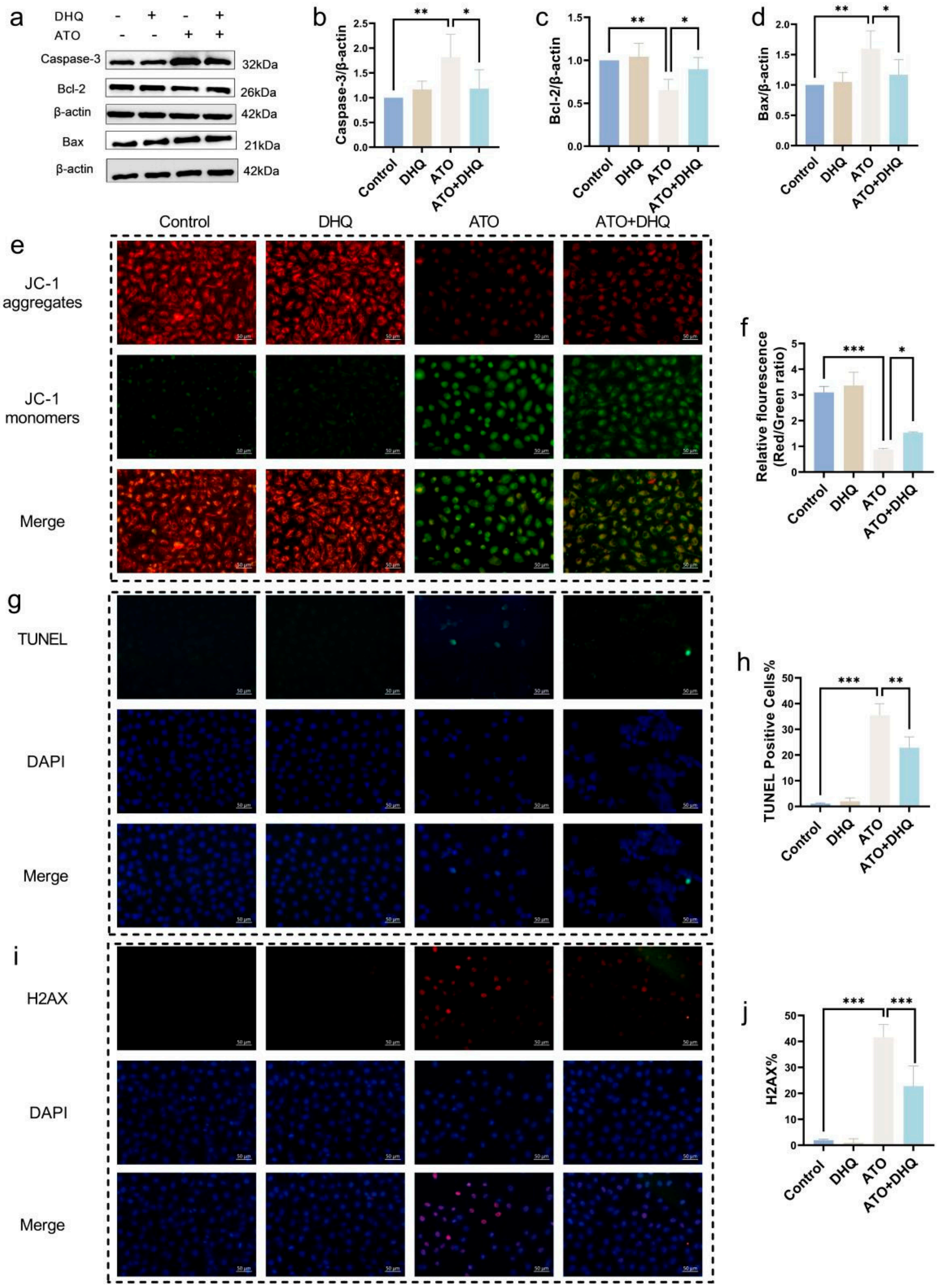

Figure S1. DHQ attenuates ATO-induced cell apoptosis in HUVECs.

(a) Western blotting analysis of Caspase-3, Bax, and Bcl-2 in HUVECs. **(b-d)** Quantitative analysis of Bax/β-actin (n=5), Bcl-2/β-actin (n=5), and Caspase-3/β-actin (n=5) in HUVECs. **(e)** Representative fluorescence images of JC-1 staining. Scale bar = 50 μm (magnification: 20×). **(f)** Quantitative analysis of the relative fluorescence (red/green ratio) in HUVECs. **(g)** Representative fluorescence images of TUNEL staining. Scale bar = 50 μm (magnification: 20×). **(h)** Quantitative analysis of TUNEL-positive cells in HUVECs. **(i)** Representative fluorescence images of H2AX staining. Scale bar = 50 μm (magnification: 20×). **(j)** Quantitative analysis of the relative fluorescence (red/green ratio) in HUVECs.Values are

mean ± SD for each group. * $P < 0.05$, ** $P < 0.01$,*** $P < 0.001$, $n \geq 3$.

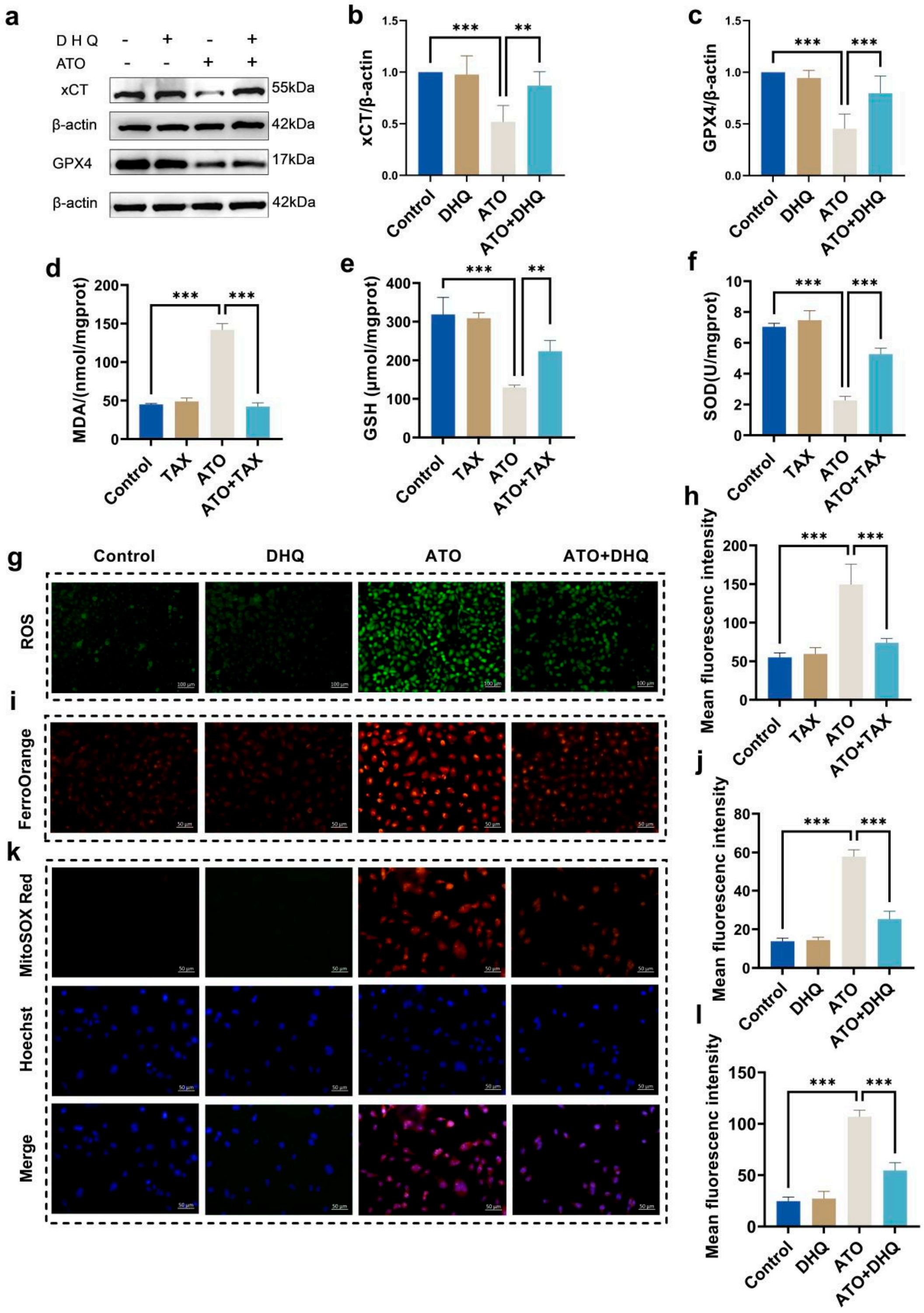

Figure S2 The effects of ATO and DHQ on ferroptosis in HUVECs

(a) Western blotting analysis of xCT and GPX4 levels in HUVECs. **(b-c)** Quantitative analysis of xCT/β-actin (n=5) and GPX4/β-actin (n=7) in HUVECs. **(d)** The MDA levels in HUVECs. **(e)** The GSH activity in HUVECs. **(f)** The SOD activity in HUVECs. **(g)** Representative fluorescence images of ROS staining in HUVECs. Scale bar = 100 μm (magnification:

10×). **(h)** Quantitative analysis of ROS fluorescence intensity in HUVECs. **(i)** Representative fluorescence images of iron ion staining in HUVECs. Scale bar = 50 μm (magnification: 20×). **(j)** Quantification of the fluorescence intensity of iron ions in HUVECs. **(k)** Representative fluorescence images of MitoSOX. Scale bar = 50 μm (magnification: 20×). **(l)** Quantification of the fluorescence intensity of MitoSOX in HUVECs.Values are mean ± SD for each group.* *P < 0.05, **P < 0.01, *** P < 0.001, n≥3.*

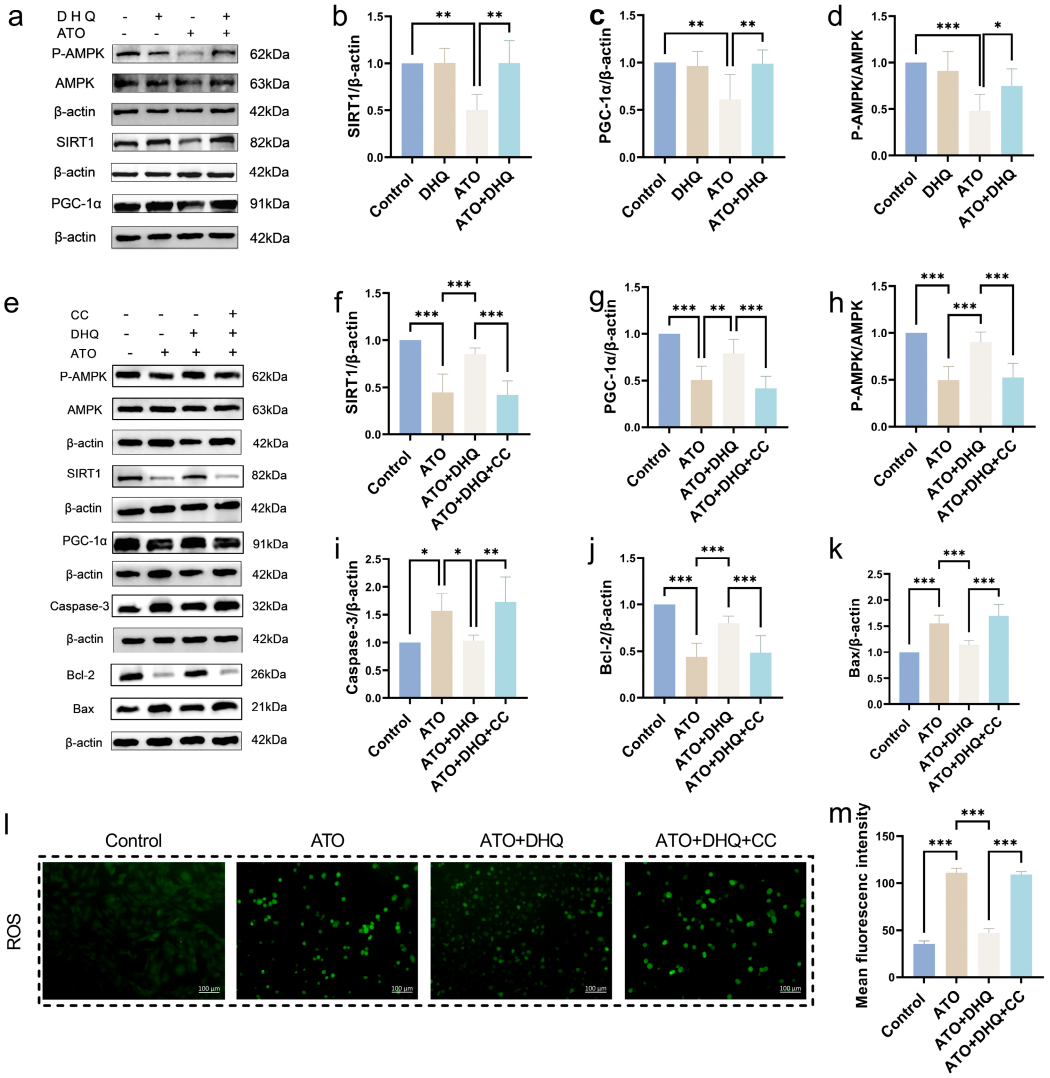

Figure S3 DHQ ameliorates ATO-induced and apoptosis by activating AMPK/SIRT1/PGC-1*α* signaling pathway in HUVECs

(a) Western blotting analysis of P-AMPK, AMPK, SIRT1 and PGC-1*α* levels in HUVECs. **(b-d)** Quantitative analysis of P-AMPK/AMPK (n=5), SIRT1/β-actin (n=5) and PGC-1*α*/β-actin (n=6) in HUVECs. **(e)** Western blotting analysis of P-AMPK, AMPK, SIRT1, PGC-1*α*, Caspase-3, Bcl-2, and Bax levels in HUVECs. **(f-k)** Quantitative analysis of P-AMPK/AMPK (n=5), SIRT1/β-actin (n=5), PGC-1*α*/β-actin (n=7), Bax/β-actin (n=6), Bcl-2/β-actin (n=8), and Caspase-3/β-actin (n=6) in HUVECs. **(l-m)** Representative fluorescence images of ROS staining in HUVECs. Scale bar = 100 μm (magnification: 10×). Values are mean ± SD for each group. ** P < 0.05, **P < 0.01, *** P < 0.001, n≥3.*

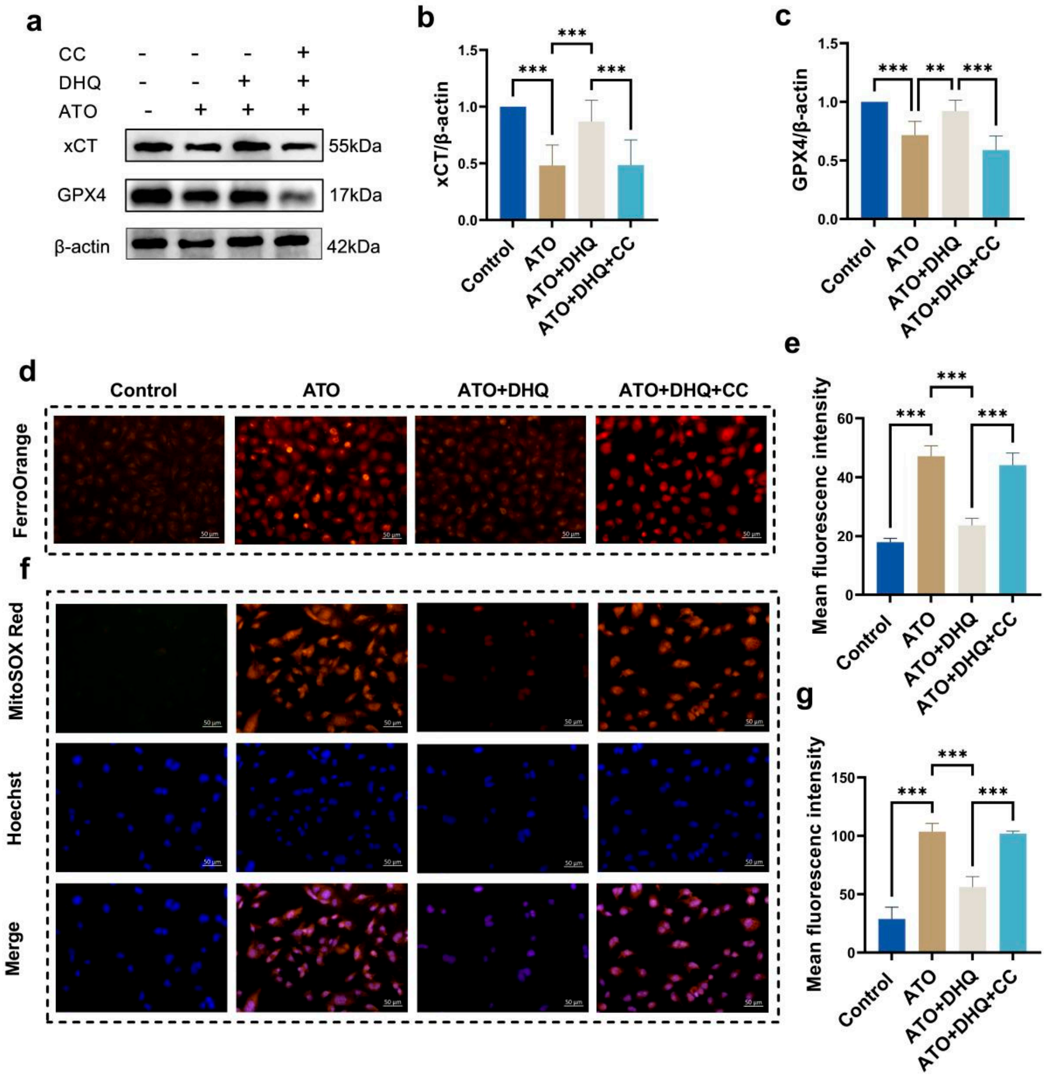

Figure S4 DHQ meliorated ATO-induced ferroptosis by the AMPK/SIRT1/PGC-1α signaling pathway in HUVECs

(a) Western blotting analysis of GPX4 and xCT levels in HUVECs. **(b-c)** Quantitative analysis of xCT/β-actin (n=9) and GPX4/β-actin (n=6) in HUVECs. **(d)** Representative fluorescence images of iron ion staining in HUVECs. Scale bar = 50 μm (magnification: 20×). **(e)** Quantification of the fluorescence intensity of iron ions in HUVECs. **(f)** Representative fluorescence images of MitoSOX. Scale bar = 50 μm (magnification: 20×). **(g)** Quantitative analysis of the relative fluorescence in HUVECs. Values are mean ± SD for each group. *$^{**}P < 0.01$, $^{***} P < 0.001$, $n \geq 3$.*

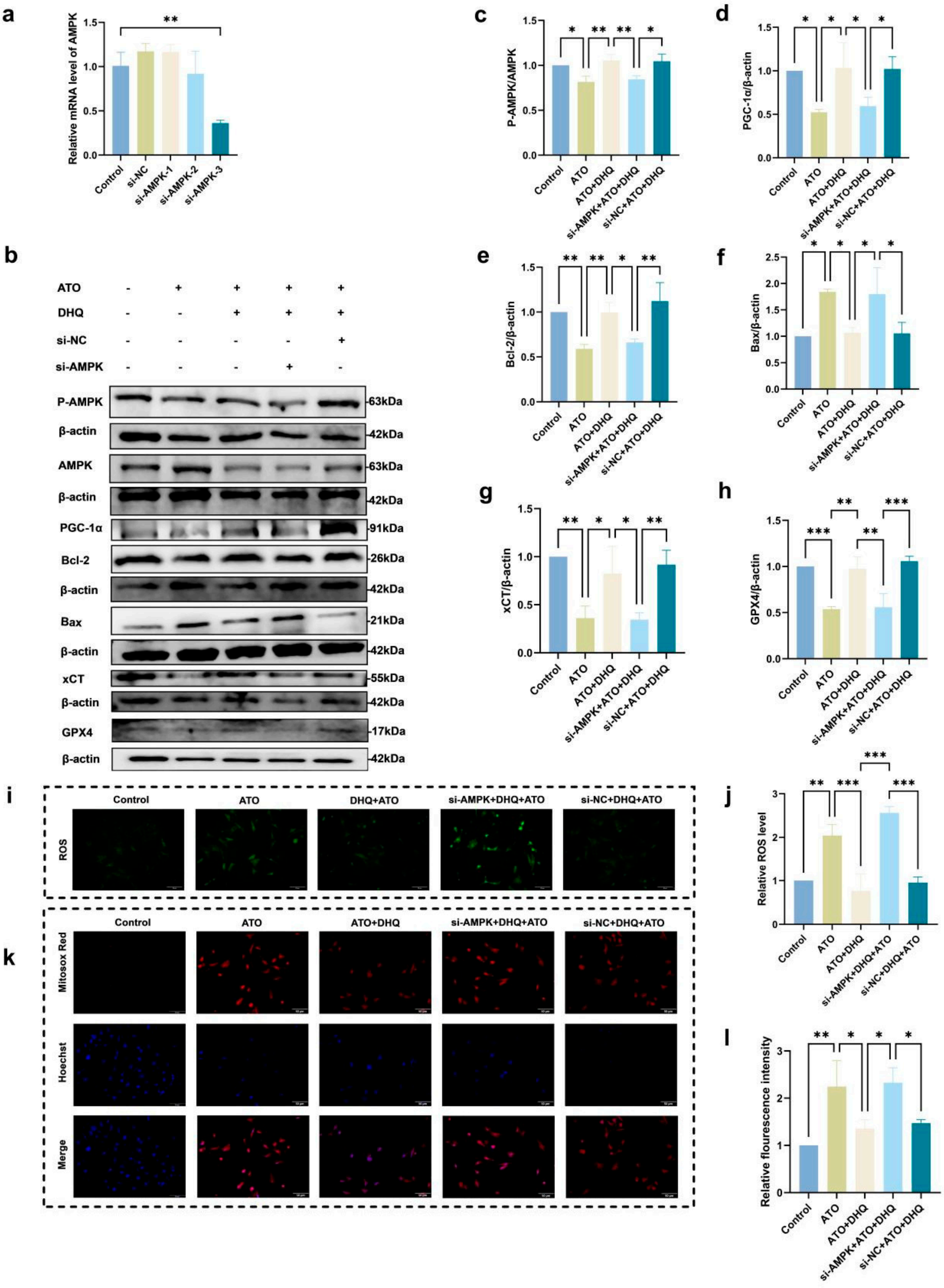

Figure S5 DHQ ameliorates ATO-induced apoptosis by activating AMPK/SIRT1/PGC-1α signaling pathway in the primary cardiomyocytes of neonatal mice.

(a) qRT-PCR analysis of AMPK mRNA expression in primary mouse cardiomyocytes. (b) Western blotting analysis of P-AMPK, AMPK, PGC-1α, Bcl-2, Bax, xCT and GPX4 levels in primary mouse cardiomyocytes. (c-h) Quantitative analysis of P-AMPK/AMPK (n=3), PGC-1α/β-actin (n=3), Bcl-2/β-actin (n=3), Bax/β-actin

(n=3), xCT/β-actin (n=3) and GPX4/β-actin (n=3) in primary mouse cardiomyocytes. (i) Representative fluorescence images of ROS staining in primary mouse cardiomyocytes. Scale bar = 50 μm (magnification: 20×). (j) Relative ROS level in primary mouse cardiomyocytes. (k) Representative fluorescence images of MitoSOX. Scale bar = 50 μm (magnification: 20×). (l) Relative fluorescence intensity of MitoSOX in primary mouse cardiomyocytes. Values are mean ± SD for each group.* $P < 0.05$, **$P < 0.01$, *** $P < 0.001$, n=3.
